# Epidemiology and outcomes of Carbapenem-resistant Enterobacterales infection in high-risk patients in Saudi Arabia

**DOI:** 10.3389/fmicb.2025.1619611

**Published:** 2025-08-19

**Authors:** Basem M. Alraddadi, Emily L. G. Heaphy, Reem Almaghrabi, Sahar Althawadi, Ahmad M. Alshahrani, Salma Almosallam, Abdullah Alraddadi, Lama Hefni, Mooza S. Almannaei, Ibrahim Bahabri, Rabab Taha, Ebtihal Alamoudi, Mohammed Bosaeed

**Affiliations:** ^1^Medicine Department, King Faisal Specialist Hospital and Research Center, Jeddah, Saudi Arabia; ^2^Medicine Department, Al Faisal University, Riyadh, Saudi Arabia; ^3^Research-Jeddah Department, King Faisal Specialist Hospital and Research Center, Jeddah, Saudi Arabia; ^4^Organ Transplant Center of Excellence, King Faisal Specialist Hospital and Research Center, Riyadh, Saudi Arabia; ^5^Pathology and Laboratory Medicine Department, King Faisal Specialist Hospital and Research Center, Jeddah, Saudi Arabia; ^6^King Faisal Specialist Hospital and Research Center, Jeddah, Saudi Arabia; ^7^Medicine Department, King Abdulaziz Medical City, Riyadh, Saudi Arabia; ^8^Medicine Department, International Medical Center, Jeddah, Saudi Arabia

**Keywords:** CRE genes, epidemiology, bone marrow transplant (BMT), solid organ transplant (SOT), Enterobacterales

## Abstract

**Introduction:**

Solid organ transplant (SOT) recipients, bone marrow transplant (BMT) recipients, and patients with hematological malignancies experience increased morbidity due to infections caused by multidrug-resistant, Carbapenem-resistant Enterobacterales (CRE). The current study aimed to further describe the epidemiology and outcomes associated with CRE infection in high-risk SOT and BMT recipients and in patients with hematological malignancies in Saudi Arabia.

**Methods:**

Patients aged 16 years and older admitted to a participating hospital between October 2018 and August 2024 who received an SOT or a BMT or were diagnosed with a hematological malignancy and had a confirmed CRE infection were included in this retrospective cohort study. A total of 155 eligible patients were included in the study population. The primary outcome of interest was all-cause mortality within 90 days of the date of the first CRE culture.

**Results:**

Among the 155 patients, 118 (76.1%) had received a solid organ transplant, while 37 (23.9%) had either a bone marrow transplant or a hematological malignancy. The BMT recipients and patients with hematological malignancies were 2.84 times more likely to die within 90 days of their first positive culture [adjusted odds ratio (AOR) = 2.84, 95% confidence interval (CI) = 1.01–8.01, *p* = 0.049]. Compared to the patients with CRE infections carrying the *bla*NDM gene, after controlling for other predictors, patients with infections harboring the *bla*OXA-48 gene were 3.97 times more likely to die within 90 days of the first culture (AOR = 3.97, 95%CI = 1.04–15.15, *p* = 0.044).

**Conclusion:**

The BMT recipients and patients with CRE infections harboring the blaOXA-48 gene were at greater risk for 90-day all-cause mortality in Saudi Arabia, confirming previous findings of high mortality rates associated with CRE infections in immunocompromised populations.

## Introduction

Bloodstream infections and other frequently encountered infections in clinical practice, such as pneumonia and complicated urinary tract infections (UTIs), caused by Carbapenem-resistant Enterobacterales (CRE) are associated with increased morbidity and mortality (Wright et al., 2021). More recently, global dissemination of CRE has occurred at an alarmingly rapid pace (Logan and Weinstein, 2017). Internationally, the focus has shifted toward early detection and infection control (Logan and Weinstein, 2017; Patel and Bonomo, 2013). CRE have become endemic in some regions of Europe, Asia, South America, and Africa, with high-risk patients, such as solid organ transplant (SOT) recipients, bone marrow transplant (BMT) recipients, and patients with hematological malignancies, being particularly vulnerable to infection due to frequent therapy with antibiotics and prolonged hospital stays (Pouch and Satlin, 2017; Nordmann et al., 2011).

Solid organ transplant recipients, BMT recipients, and patients with hematological malignancies have experienced increasing morbidity due to infections caused by multidrug-resistant CRE (Kerneis and Lucet, 2019; Patel et al., 2008). Approximately 1.8–2.0% of BMT recipients experienced incident bloodstream infections caused by CRE, and the 30-day mortality rates ranged from 52 to 63% following bacteremia in patients with hematological malignancies (Pouch and Satlin, 2017; Yang et al., 2020). Approximately one-quarter (range of 16–24%) of all patients with CRE bacteremia are patients with hematological malignancies (Pouch and Satlin, 2017).

The incidence of CRE infection among SOT recipients ranges from 3.0 to 23%, and overall mortality rates associated with CRE infection range from 24 to 70% (Freire et al., 2019; Aguado et al., 2018; Gutiérrez-Gutiérrez et al., 2017; Tzouvelekis et al., 2012). Although a recent matched cohort study of patients with CRE found that SOT patients with CRE did not have worse outcomes than non-transplant patients with CRE, 90-day mortality remained substantial among the SOT patients. The study concluded that reducing hospital stay to prevent CRE infection should be a goal for transplant programs (Boutzoukas et al., 2025). Overall, data on patients with CRE infections and immunosuppression remain limited.

A previous study conducted in Saudi Arabia on hospitalized patients showed that OXA-48 and NDM carbapenemases are the most common enzymes found in CRE infections caused by *Klebsiella pneumoniae* (Alraddadi et al., 2022). Both this multicenter prospective study and a more recent retrospective cohort study conducted in the region provided specific details regarding CRE colonization and infection in high-risk patients (Alraddadi et al., 2022; Alraddadi et al., 2024). The current study aimed to further describe the epidemiology and outcomes associated with CRE infection in high-risk SOT patients, BMT recipients, and patients with hematological malignancies in Saudi Arabia.

## Materials and methods

### Study design and patient population

Patient data were collected from King Faisal Specialist Hospital & Research Center (KFSH&RC) in Riyadh (*n* = 73), KFSH&RC in Jeddah (*n* = 38), and King Abdullah International Medical Research Center (KAIMRC) in Riyadh (*n* = 44) between October 2018 and August 2024 for this retrospective cohort study. These tertiary care medical centers were included because they are leading centers in Saudi Arabia in terms of both quality of care and the number of SOT and BMT cases. Adult patients aged 16 years and older admitted to the hospital who received a solid organ transplant or a bone marrow transplant or were diagnosed with a hematological malignancy and had a confirmed CRE infection were included, resulting in a total study population of 155 patients. The primary outcome of interest was all-cause mortality within 90 days of the date of the first CRE culture.

### Microbiological procedures

After obtaining the culture, the Vitek 2 system (bioMérieux, Marcy L’étoile, France) was used to perform bacterial identification and susceptibility testing as phenotypic methods for detecting CRE, following the methodology from the Clinical and Laboratory Standards Institute (M-100, 33rd Edition). The Cepheid Xpert Carba-R assay (Cepheid, Sunnyvale, CA, USA) is an automated *in vitro* diagnostic test for the qualitative detection of the presence of *bla*_KPC_*, bla*_NDM_*, bla*_VIM_*, bla*_OXA-48_, and *bla*_IMP_ gene sequences. It differentiates these carbapenem-resistance–associated sequences in Gram-negative bacteria and provides a non-detected result for other sequences (Bianco et al., 2022). The Xpert Carba-R assay was used to test confirmed isolates from the culture, following recommended procedures to detect and differentiate *bla*_KPC_*, bla*_NDM_*, bla*_VIM_*, bla*_OXA-48_, and *bla*_IMP_ genes.

### Data collection and statistical analysis

Information about demographic and clinical characteristics and patient outcomes was abstracted from medical records at the three hospitals. Data were collected for the following variables: date of birth, sex, comorbid conditions, Charlson Comorbidity Index, APACHE II score, Pitt bacteremia score, 30-day history of intensive care unit (ICU) stay and procedures, prior CRE colonization and infection status within 90 days, isolated organism, susceptibility to antibiotics, type of CRE infection, and antibiotic treatment course. These variables were considered potential predictors of 90-day all-cause mortality. In addition to mortality, data were collected on relapse within 30 days, microbiological eradication (defined as documentation of subsequent cultures showing infection clearance, confirmed by negative results), CRE-attributable mortality, acute kidney injury, and clinical cure. CRE-attributable mortality was defined as in-hospital death clearly linked to a CRE infection, with no other obvious cause identified.

Continuous predictor variables were assessed for normality by examining the ratio of mean to median values, evaluating skewness and kurtosis, applying the Kolmogorov–Smirnov test, and by observing the data graphically. Mean and standard deviation (SD) were reported for normally distributed variables, while median and interquartile range (IQR) were reported for non-normally distributed variables. Frequencies were generated for categorical variables. Associations between predictor variables and 90-day all-cause mortality were assessed using Student’s *t*-test for normally distributed data, the Wilcoxon two-sample test for non-parametrically distributed continuous variables, and the chi-squared and Fisher’s exact test for categorical data. All predictor variables found to be significantly associated with mortality were included in a multivariable logistic model, along with age, to predict 90-day all-cause mortality. A separate model was also created to examine these associations in the patients with bacteremia (*n* = 72). The analyses were performed using SAS Studio, with the significance level set at *α* = 0.05. The study was approved by the Institutional Review Board of the KFSH&RC in Jeddah.

## Results

The study population for this retrospective study comprised 155 patients with CRE infections, of whom 118 (76.1%) had received a solid organ transplant (SOT) and 37 (23.9%) had a bone marrow transplant (BMT) or had a hematological malignancy (Table 1). There were 64 (54.2%) patients who received kidney transplants, 42 (35.6%) patients who received liver transplants, one (0.9%) patient who received a heart transplant, 13 (11.0%) patients who received a lung transplant, and two (1.7%) patients who received a small bowel/intestine transplant. In addition, four patients received both kidney and liver transplants. The median [interquartile range (IQR)] age was 53.5 (38.1–64.4) years, and 56.8% of the population was male. The most common comorbidities among the patients were diabetes mellitus (41.9%) and hypertension (40.0%) (Table 1).

**Table 1:** Demographic and clinical characteristics of the study population at baseline (*n* = 155).

Variable	*n* (%)	Mean ± SD/ Median (IQR)^a^
Age (years)		53.5 (38.1–64.4)
Sex		
Male	88 (56.8)	
Female	67 (43.2)	
Patient type		
BMT and hematological malignancy	37 (23.9)	
Allogenic	15 (40.5)	
Autologous	4 (10.8)	
No transplant	18 (48.7)	
SOT^b^	118 (76.1)	
Kidney	64 (54.2)	
Liver	42 (35.6)	
Heart	1 (0.9)	
Lung	13 (11.0)	
Small bowel/intestine	2 (1.7)	
Comorbidities		
IHD	14 (9.0)	
HF	19 (12.3)	
PVD	3 (1.9)	
CVA	10 (6.5)	
CKD	23 (14.8)	
ESRD	24 (15.5)	
HIV	0 (0.0)	
Connective tissue disease	6 (3.9)	
Liver disease	38 (24.5)	
DM	65 (41.9)	
Solid tumor	4 (2.6)	
Primary immunodeficiency	2 (1.3)	
HTN	62 (40.0)	
Charlson Comorbidity Index		3 (2–6)
Previous colonization with CRE (within 90 days)	26 (16.8)	
In the 30 days prior to CRE positive culture		
ICU stay	69 (44.5)	
Endotracheal tube	60 (38.7)	
Surgery	49 (31.6)	
Carbapenem exposure	91 (58.7)	
Central line	94 (60.7)	
Type of CRE infection^c^		
Central line-associated bloodstream infection	25 (16.1)	
HAP/VAP	30 (19.4)	
UTI	50 (32.3)	
Complicated intra-abdominal infection	41 (26.5)	
Skin and soft tissue infection	10 (6.5)	
Joint/bone infection	0 (0.0)	
Primary bacteremia (source unidentified)	8 (5.2)	
Other	12 (7.7)	
Organism isolated		
*E. coli*	36 (23.2)	
*K. pneumoniae*	119 (76.8)	
Molecular type of CRE infection^d^		
blaKPC	4 (2.6)	
blaNDM	51 (32.9)	
blaIMP	1 (0.7)	
blaOXA-48	89 (57.4)	
None detected	14 (9.0)	
Not available	20 (12.9)	
CRE bacteremia	72 (46.5)	
Pitt bacteremia score (*n* = 72)		2.0 (0.0–5.5)
APACHE II Score (*n* = 65)		24.2 ± 11.7
Definitive therapy for CRE		
Meropenem	24 (15.5)	
Ceftazidime/avidactam	105 (67.7)	
Imipenem-cilastatin-relebactam	4 (2.6)	
Imipenem	2 (1.3)	
Aztreonam	43 (27.7)	
Gentamicin	12 (7.7)	
Amikacin	8 (5.2)	
Colistin	20 (12.9)	
Tigecycline	16 (10.3)	
Fosfomycin	2 (1.3)	
Cefedricol	2 (1.3)	
Tobramycin	0 (0.0)	
Ertapenem	0 (0.0)	
Bactrim	5 (3.2)	
Ciprofloxacin	1 (0.7)	
Levofloxacin	0 (0.0)	
Nitrofurantoin	1 (0.7)	

SD, standard deviation; IQR, Interquartile Range; BMT, bone marrow transplantation; SOT, solid organ transplantation; IHD, ischemic heart disease; HF, heart failure; PVD, peripheral vascular disease; CVA, cerebral vascular accident; CKD, chronic kidney disease; ESRD, end-stage renal disease; HIV, human immunodeficiency virus; DM, diabetes mellitus; HTN, hypertension; CRE, Carbapenem-resistant Enterobacterales; ICU, intensive care unit; HAP/VAP, hospital-acquired pneumonia/ventilator-associated pneumonia; UTI, urinary tract infection.

a Reported according to the distribution of the data.

b Four patients received kidney and liver transplants.

c More than one CRE infection type was possible per patient.

d More than one CRE molecular type was possible per patient; percentages may total more than 100 due to rounding.

While 16.8% of the patients had documented CRE colonization within the last 90 days, almost one-third of the patients (32.3%) had a UTI (Table 1). The median (IQR) Charlson Comorbidity Index was 3 (2–6). Among the 72 (46.5%) patients with CRE bacteremia and a Pitt bacteremia score, the median score was 2.0 (0.0–5.5). More than three-quarters of the isolated organisms (76.8%) were *K. pneumoniae,* and the *bla*_OXA-48_ (57.4%) gene was the most common molecular type, followed by *bla*_NDM_ (32.9%).

Following the hospital protocol for treatment, definitive therapy for CRE infection included ceftazidime/avibactam for 67.7% of the patients, while 27.7% received aztreonam. The majority of the patients with the *bla*_OXA-48_ gene received ceftazidime/avibactam, while those with the *bla*_NDM_ gene were treated with a combination of ceftazidime/avibactam and aztreonam (Table 1). Among the 61 patients with available sensitivity data for ceftazidime/avibactam and the *bla*OXA-48 molecular type, 17 (27.9%) showed resistance to this treatment regimen, while 44 patients (72.1%) had susceptible isolates. However, when analyzing the subgroup of 43 patients with only the *bla*OXA-48 gene, only two patients (4.7%) demonstrated resistance to ceftazidime/avibactam. The median (IQR) MIC value for ceftazidime/avibactam for patients with NDM and patients with OXA-48 was 16 (1–16).

There were 64 patients (41.3%) admitted to the intensive care unit (ICU) during their hospital stay, and over one-third of all patients (36.1%) experienced acute kidney injury (Table 2). Just over one-third (34.2%) of patients died within 90 days of their first CRE culture, with 67.9% of these deaths being attributable to CRE (Table 2). Attributable mortality was defined as death directly linked to CRE infection, including cases with no other clear cause of death or cases where the patient had persistent infection without clinical improvement until death.

**Table 2:** Outcomes of the study population (*n* = 155).

Variable	*n* (%)
Admitted to ICU	64 (41.3)
Relapse within 30 days	32 (20.7)
Acute kidney injury	56 (36.1)
Microbiological eradication	88 (56.8)
Death within 90 days of the first culture	53 (34.2)
CRE Attributable mortality^b^	36 (67.9)
Clinical cure	115 (74.2)

ICU, intensive care unit; CRE, Carbapenem-resistant Enterobacterales.

b Out of 53 patients who died.

The association between patient type and mortality was significant in this patient population, where 34.0% of the patients who died were BMT recipients compared to 18.6% of survivors who were BMT recipients (*p* = 0.03) (Table 3). Among the patients who died, 35.9% had hospital-acquired pneumonia/ventilator-associated pneumonia (HAP/VAP), compared to 10.8% of the patients who survived with this infection (*p* < 0.001). The association between the type of carbapenemase found in the CRE isolates from the patients’ infections and mortality was also significant. Among the deaths, 75.5% (*n* = 53) occurred in the patients with isolates harboring *bla*NDM, *bla*OXA-48, or both *bla*NDM and *bla*OXA-48. While 62.3% of the patients who died developed CRE bacteremia, 38.2% of survivors were bacteremic (*p* = 0.003) (Table 3).

**Table 3:** Univariate analyses of the associations between demographic/clinical variables and mortality.

Variable	Mortality^a^		*p*-value
	Yes	No	
	(*n* = 53)	(*n* = 102)	
Age (years)	56.7 (47.6–64.0)	51.5 (37.5–64.7)	0.34
Sex			
Male	32 (60.4)	56 (54.9)	
Female	21 (39.6)	46 (45.1)	0.61
Patient type			
BMT	18 (34.0)	19 (18.6)	
SOT	35 (66.0)	83 (81.4)	0.03
Type of BMT patient (*n* = 37)			
Allogenic	8 (44.4)	7 (36.8)	
Autologous	0 (0.0)	4 (21.1)	0.16
No transplant	10 (55.6)	8 (42.1)	
Type of SOT transplant (*n* = 118)			
Kidney	14 (40.0)	46 (55.4)	
Liver	15 (42.9)	23 (27.7)	
Heart	0 (0.0)	1 (1.2)	0.34
Lung	3 (8.6)	10 (12.1)	
Small bowel/intestine	1 (2.9)	1 (1.2)	
Kidney and liver (*n* = 4)	2 (5.7)	2 (2.4)	
Charlson comorbidity Index	4 (2–6)	3 (2–6)	0.30
Type of CRE infection			
CLABSI	12 (22.6)	13 (12.8)	0.17
HAP/VAP	19 (35.9)	11 (10.8)	<0.001
UTI	8 (15.1)	42 (41.2)	0.001
Complicated intra-abdominal infection	14 (26.4)	27 (26.5)	0.99
Skin and soft tissue infection	4 (7.6)	6 (5.9)	0.74
Primary bacteremia (source unidentified)	2 (3.8)	6 (5.9)	0.72
Other	6 (11.3)	6 (5.9)	0.34
Organism isolated			
*E. coli*	8 (15.1)	28 (27.5)	
*Klebsiella*	45 (84.9)	74 (72.6)	0.08
Molecular typing common genes (*n* = 118 infections)			
blaNDM	4 (10.0)	25 (32.1)	
blaOXA-48	29 (72.5)	38 (48.7)	
blaNDM and blaOXA-48	7 (17.5)	15 (19.2)	0.02
CRE bacteremia	33 (62.3)	39 (38.2)	0.003
Pitt bacteremia score (*n* = 72)	4.0 (1.0–8.0)	2.0 (0.0–3.0)	0.01
APACHE II Score (*n* = 65)	26.5 ± 12.1	20.7 ± 10.2	0.05
Relapse within 30 days	13 (24.5)	19 (18.6)	0.39
Acute kidney injury	31 (58.5)	25 (24.5)	< 0.001

CRE, Carbapenem-resistant Enterobacterales; HAP/VAP, hospital-acquired pneumonia/ventilator-associated pneumonia; UTI, urinary tract infection.

a Values are presented according to the statistical test performed, with median (IQR) for non-normally distributed variables (Wilcoxon two-sample test), mean ± SD for normally distributed variables (Student’s *t*-test), and *n* (%) for chi-squared/Fisher’s exact tests; percentages may total more than 100 due to rounding.

Predicting mortality using a multivariable logistic regression model indicated that the BMT recipients and patients with hematological malignancies were 2.84 times more likely to die within 90 days of their first positive culture compared to the SOT recipients [adjusted odds ratio (AOR) = 2.84, 95% confidence interval (CI) = 1.01–8.01, *p* = 0.049] (Table 4). The participants who developed HAP/VAP were also significantly more likely to experience 90-day all-cause mortality (AOR = 3.70, 95% CI = 1.33–10.30, *p* = 0.012). Compared to the patients with the *bla*NDM gene, the patients with an infection harboring the *bla*OXA-48 gene were 3.97 times more likely to die within 90 days of the first culture (AOR = 3.97, 95%CI = 1.04–15.15, *p* = 0.044). Having CRE bacteremia and acute kidney injury were also associated with an increased likelihood of experiencing 90-day all-cause mortality (Table 4). A multivariable model analyzing the patients with CRE bacteremia (*n* = 72) revealed that the BMT recipients (*n* = 29 of the 72, 40.3%) had an increased likelihood of experiencing 90-day mortality compared to the SOT recipients (AOR = 3.71, 95% CI = 1.05–13.14, *p* = 0.042). In addition, for every one-unit increase in the Pitt bacteremia score, the patients were 1.32 times more likely to die within 90 days of their first positive culture (*p* = 0.005) (Table 5).

**Table 4:** Multivariable analysis predicting mortality by predictor variables (*n* = 155).

Predictor	OR	95% CI	*p*-value
Age (years)	1.03	(1.00–1.06)	0.062
Patient type (BMT)	2.84	(1.01–8.01)	0.049
CRE bacteremia	2.52	(1.00–6.34)	0.049
HAP/VAP infection	3.70	(1.33–10.30)	0.012
UTI	0.42	(0.15–1.18)	0.100
Molecular typing (ref = blaNDM)			
blaKPC, blaIMP, none detected/done	3.08	(0.71–13.27)	0.132
blaOXA-48	3.97	(1.04–15.15)	0.044
blaNDM and blaOXA-48	2.58	(0.54–12.46)	0.238
Acute kidney injury	3.77	(1.64–8.70)	0.002

HAP/VAP, hospital-acquired pneumonia/ventilator-associated pneumonia; UTI, urinary tract infection; OR, odds ratio; CI, confidence interval.

**Table 5:** Multivariable analysis predicting mortality by predictor variables in the bacteremic patients (*n* = 72).

Predictor	OR	95% CI	*p*-value
Patient type (BMT)	3.71	(1.05–13.14)	0.042
HAP/VAP infection	1.66	(0.30–9.27)	0.566
UTI	0.41	(0.10–1.80)	0.239
Acute kidney injury	2.52	(0.80–7.96)	0.116
Pitt bacteremia score	1.32	(1.09–1.61)	0.005

HAP/VAP, hospital-acquired pneumonia/ventilator-associated pneumonia; UTI, urinary tract infection; OR, odds ratio; CI, confidence interval.

## Discussion

This study reveals that mortality is alarmingly high among immunosuppressed SOT patients and BMT recipients, with more than one-third of the population dying within 90 days of their first positive culture and just over two-thirds of these deaths being attributable to CRE infection. Furthermore, BMT recipients, patients with hematological malignancies, and patients infected with CRE carrying the *bla*OXA-48 gene are at increased risk of 90-day mortality. Given the high prevalence of blaOXA-48 CRE infections in the region, this is particularly concerning due to the heightened risk these high-risk patients face of developing infections, compounded by recurrent antibiotic use and prolonged hospital stays. Despite the availability of effective antibiotics for CRE, it is alarming that mortality in this patient population remains high. This is likely due to delays in initiating the appropriate antibiotic therapy or the development of resistance. These factors highlight the urgent need for rapid diagnostic tools with quick turnaround times to detect CRE and the current unmet needs regarding the limited treatment options for CRE.

Due to sample size considerations, the SOT patients and the BMT recipients were combined in the analyses. Overall, 29.7% of the SOT patients died compared to 48.6% of the BMT recipients, which was not unexpected considering the severity of illness among many BMT recipients. While more than three-quarters of all patients had a *K. pneumoniae* infection, 37.8% of these patients died, compared to 22.2% of the patients with an *E. coli* infection. Running the multivariable model with only the SOT patients revealed similar findings, except that the association with 90-day mortality was no longer significant for the patients with the *bla*OXA-48 isolate or for the bacteremic patients. When the lung, heart, and small bowel SOT patients (*n* = 16) were removed from the SOT model, the results were similar to the original model, except that the association between *bla*OXA-48 isolates and mortality was no longer significant.

A matched case–control study compared patients with carbapenem-resistant *K. pneumoniae* to patients with carbapenem-susceptible *K. pneumoniae* infections as controls (Patel et al., 2008). Carbapenem-resistant *K. pneumoniae* infection was found to be associated with recent organ or stem-cell transplantation, and the cases were more likely than the controls to die during hospitalization and from the infection (Patel et al., 2008). SOT patients have increased susceptibility to infections related to long-term immunosuppression. Although there are specific guidelines, it is still challenging to determine how this condition can be managed in recipients with CRE infections (Pérez-Nadales et al., 2022).

Carbapenem resistance has been shown to be a threat to allograft and patient survival, with limited antibiotics available for treatment (Patel et al., 2010). Among SOT recipients in CRE-endemic areas, 3.0–10% develop infections correlated with the transplanted organ (Satlin et al., 2014). Active surveillance is needed to monitor and prevent infection in immunocompromised patients, especially considering that empirical therapy is not active against CRE and its identification can take 2–4 days (Satlin et al., 2014; Blaschke et al., 2012). In addition, several studies have revealed high in-hospital mortality rates for CRE bacteremia among greatly immunocompromised BMT recipients and patients with hematological malignancies, with the majority of deaths being attributable to the infection (Satlin et al., 2014; Satlin et al., 2013; Muchtar et al., 2012; Snitkin et al., 2012). Analyses from a study indicated that considering genomic and epidemiological data simultaneously may assist in controlling CRE hospital-acquired infections (Snitkin et al., 2012). More information is needed regarding factors that may be specific to immunocompromised patients to facilitate CRE prevention in hospitals (Satlin et al., 2014).

The results from our study revealed that a number of factors were independently associated with 90-day all-cause mortality, including the type of patient (BMT compared to SOT), bacteremia, hospital-acquired pneumonia/ventilator-associated pneumonia (HAP/VAP), CRE infection carrying *bla*OXA-48, and acute kidney injury. A multivariable model for the bacteremic patients (*n* = 72) indicated that being a BMT patient (reference SOT) and a higher Pitt bacteremia score were significant predictors of mortality.

A recent study with some similar findings reported that 30-day mortality of CRE infection was independently associated with higher Simplified Acute Physiology Score II, sepsis at the time of diagnosis, pneumonia, monotherapy, and inappropriate empiric antibiotic therapy (So-Ngern et al., 2023). Additional studies have shown that factors associated with 14-, 28-, and 30-day mortality, infection-related mortality, and overall hospital mortality in patients with CRE infections include chronic renal failure, high APACHE II score, presentation with septic shock and severe sepsis, neutropenia, dialysis, age over 60 years, ultimately fatal disease, and rapidly fatal underlying diseases (Li et al., 2019; Tumbarello et al., 2015; de Maio Carrilho et al., 2016; Daikos et al., 2014). In our study, we were not able to determine why patients with infections caused by CRE-producing OXA-48 were more likely to die compared to those infected with other carbapenemase-producing strains. It is not clear whether this is due to the development of further antimicrobial resistance or the associations with other types of resistance genes. The most plausible explanation is the presence of unmeasured confounders not captured in our analysis, most likely related to the timing of antibiotic initiation and the appropriateness of the empirical regimen for this specific carbapenemase enzyme.

The current study benefited from the inclusion of a decent sample size of immunocompromised patients with molecular testing for CRE infection, confirming previous findings in the region regarding molecular identification for CRE infection. These studies also showed that a positive CRE screening test increased the likelihood of 30-day mortality in high-risk patients (Alraddadi et al., 2024). To date, this is the largest reported cohort of post-transplant patients with CRE in the region. In addition, our study confirmed that factors such as pneumonia and bacteremia (*n* = 72) are independently associated with mortality in patients with CRE, in line with findings from previous studies. Furthermore, we demonstrated that the BMT recipients, compared to the SOT patients, and patients with molecular identification of *bla*OXA-48 in their CRE infections were at greater risk for 90-day all-cause mortality in Saudi Arabia.

Although we were able to collect data on several potential predictor variables associated with CRE infection and mortality, the retrospective nature of our study and reliance on medical records may have limited our ability to measure or account for certain relevant factors in the analyses. It is possible that relevant clinical differences exist between BMT recipients and patients with hematological malignancies, although these patients were combined in the analysis due to sample size constraints. In addition, sequencing was not performed in this study, which may have predicted outcomes from CRE infection more accurately. The absence of sequencing may have limited our ability to detect virulence factors or resistance mechanisms that could have contributed to the observed increase in mortality. In conclusion, our study confirmed previous findings regarding the high mortality rate associated with CRE infection in immunocompromised populations. Future research should focus on developing effective strategies to care for immunocompromised patients with prolonged hospital stays to reduce the risk of acquiring CRE infections.

## Data Availability

The raw data supporting the conclusions of this article will be made available by the authors, without undue reservation.
